# Impact of Air Pollution on Lung Function among Preadolescent Children in Two Cities in Poland

**DOI:** 10.3390/jcm10112375

**Published:** 2021-05-28

**Authors:** Piotr Dąbrowiecki, Łukasz Adamkiewicz, Dominika Mucha, Piotr Oskar Czechowski, Mateusz Soliński, Andrzej Chciałowski, Artur Badyda

**Affiliations:** 1 Department of Allergology and Infectious Diseases, Military Institute of Medicine, 04-141 Warsaw, Poland; achcialowski@wim.mil.pl; 2 Polish Federation of Asthma, Allergy and COPD Patients Associations, 01-604 Warsaw, Poland; artur.badyda@pw.edu.pl; 3 Cracow Smog Alert, 31-104 Krakow, Poland; lukasz.adamkiewicz@cleanaircentre.eu (Ł.A.); dominika.mucha@pw.edu.pl (D.M.); 4 European Clean Air Center, 31-104 Krakow, Poland; 5 Faculty of Building Services, Hydro- and Environmental Engineering, Warsaw University of Technology, 00-653 Warsaw, Poland; 6 Department of Quantitative Methods and Environmental Management, Faculty of Management and Quality Science, Gdynia Maritime University, 83 Morska Street, 81-225 Gdynia, Poland; p.o.czechowski@wpit.umg.edu.pl; 7 Faculty of Physics, Warsaw University of Technology, 00-662 Warsaw, Poland; mateusz.solinski.dokt@pw.edu.pl

**Keywords:** air quality, air pollution, spirometry, lung function, children

## Abstract

Ambient air pollution impairs lung development in children, particularly in industrialized areas. The air quality in Zabrze, a city located in the Upper Silesian Industrial Region of Poland, is among the worst in Europe. We compared lung function and the frequency of respiratory or allergic symptoms between children living in Zabrze and those living in Gdynia, a city on the Baltic coast, which has the best long-term air quality in Poland. We enrolled children aged 9–15 years from both cities who were able to perform a spirometry. The following spirometry variables were measured for all participants: forced vital capacity (FVC), forced expiratory volume during the first second of expiration (FEV1), FEV1/FVC index, and peak expiratory flow (PEF). The frequencies of respiratory or allergic symptoms were taken from a survey completed by the participants’ parents. In total, 258 children from Gdynia and 512 children from Zabrze were examined. The mean values of FVC, FEV1, and PEF were significantly greater among children in Gdynia than those reported in Zabrze (*p* ≤ 0.032), and the frequencies of seasonal rhinorrhea (*p* = 0.015) or coughing episodes (*p* = 0.022) were significantly higher in Zabrze than in Gdynia. In conclusion, lung function was significantly impaired in children living in Zabrze, an area which is associated with poor air quality. Strategies to improve air quality in the Silesia region are urgently needed.

## 1. Introduction

Ambient air pollution has many detrimental health effects, causing about seven million premature deaths annually worldwide [1]. Children are particularly susceptible to the effects of ambient air pollution because their respiratory systems are immature; moreover, they are exposed to air pollution to a greater extent than adults due to having higher minute ventilation and spending more time outdoors [2]. Many studies using spirometry measures have shown that increased concentrations of different air pollutants slow down lung development in children [3,4,5,6,7,8]. In particular, children living in urbanized, industrialized areas can suffer from the effects of air pollution on lung function [9,10,11,12,13,14]. These effects are likely due to various types of air pollution, with increased concentrations of many pollutants, such as particulate matter (PM), ozone (O3), sulfur dioxide (SO2), and nitrogen dioxide (NO2) [15,16]. In addition, air pollution in children may cause cardiovascular diseases, such as hypertension, by affecting the microvasculature (arteriole constriction) [17,18].

The air quality in Silesia, an industrialized region of Poland, is among the worst in Europe. This densely populated area is responsible for most of the coal production in Poland and has numerous coal-fired power plants, steelworks, and mineral mines. Of the 50 most polluted cities in the European Union, 36 are in Poland, of which most are located in the Silesia region [19,20]. Likely due to the high level of pollution, the region of Silesia is characterized by the shortest urban life expectancy and the highest incidence of premature births, genetic birth defects, and spontaneous miscarriages in Poland [21].

Although the poor air quality in Silesia is well known, no study to date has assessed its influence on lung development in children. In this study, we compared lung function in preadolescent children living in Silesia with that of children living on the Baltic coast, which has the best long-term air quality in Poland. We used objective spirometry measures of lung function and gathered data on the frequency of respiratory and allergic symptoms.

## 2. Materials and Methods

### 2.1. Study Design and Participants

This was a cross-sectional study carried out from 16 May 2019 to 12 June 2019, among children aged 9–15 years who were capable of completing a spirometry. Children were recruited from 6 primary schools in Gdynia, Poland, and 10 primary schools in Zabrze, Poland. The study was approved by the Bioethics Committee of the Military Institute of Medicine, Warsaw, and all children and their parents agreed to take part in the study.

**Figure 1:**
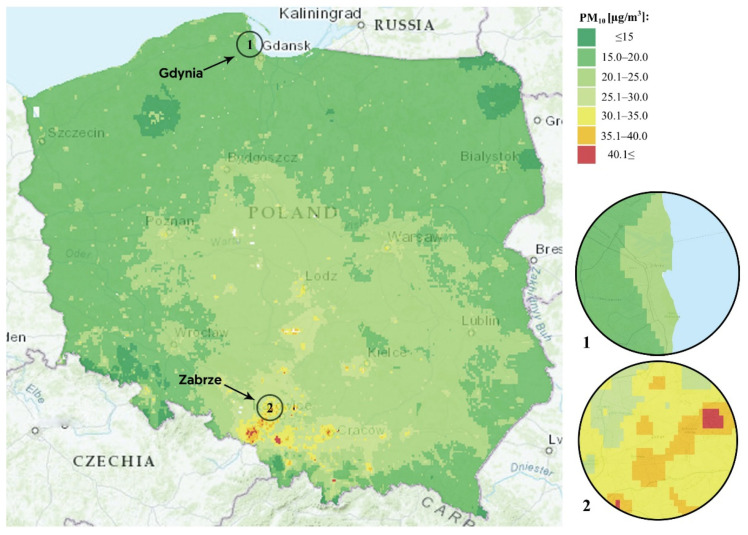
Spatial modeling of PM_10_ pollution in Poland. Inserts show Zabrze (**1**) and Gdynia (**2**).

We chose to compare Gdynia and Zabrze because these two cities differ substantially in terms of long-term air quality. Gdynia, located in the north of Poland on the Baltic coast, has good overall air quality, whereas Zabrze, located in the south of Poland in the region of Silesia, has poor overall air quality. According to the spatial modeling data of the Chief Inspectorate of Environmental Protection, the annual concentrations of PM_2.5_, PM_10_, SO_2_, and NO_2_ are higher in the whole of the Zabrze area compared to the Gdynia area, and hence the long-term air quality in any of the schools in Gdynia should be better than those in any of the schools in Zabrze. The annual mean concentrations of PM_10_ for these cities are shown in Figure 1.

We estimated whole-life exposure to air pollution for participants in Zabrze and Gdynia. Whole-life exposure to PM_10_, SO_2_, NO_2_, and O_3_ was defined as the mean of the daily concentrations for the period between the year of the participant’s birth and the start of the study (15 May 2019). Data from all available measuring stations were used—i.e., four stations in Gdynia and one station in Zabrze. The whole-life exposures were estimated to check whether the differences in air pollution between the cities remained over many years.

### 2.2. Spirometry

All spirometry examinations were conducted by trained staff with the use of an AioCare portable spirometer connected to a smartphone application (HealthUp, Warsaw, Poland) [22]. The examinations were conducted in compliance with the standards of the American Thoracic Society and the European Respiratory Society [23]. Only examinations of quality grade A or B were included in the analyses. The following spirometry variables were measured for all participants: forced vital capacity (FVC), forced expiratory volume during the first second of expiration (FEV1), FEV1/FVC index, and peak expiratory flow (PEF).

### 2.3. Survey on Respiratory Symptoms

We asked the participants’ parents to fill out a survey to gather data on the distance from their house to a major road (<100 m, 100–200 m, 200–500 m, >500 m), the use of a coal- or wood-burning stove in the house, the presence of a heating furnace in an in-house living area, the smoking status of the parents, the physical activity of the child, and the existence of an asthma diagnosis. Additionally, we asked parents about the following allergic or respiratory symptoms in their children: all-year rhinorrhea; seasonal rhinorrhea; acute allergic reactions (food, exercise, insect sting); dyspnea episodes; coughing episodes; wheezing episodes; skin changes (eczema, urticaria, edema, erythema, pruritus); allergies (any); allergic skin reactions; recurring bronchitis with coughing, wheezing, or dyspnea; and wheezing (ever).

### 2.4. Statistical Analysis

Descriptive statistics were chosen based on data distribution. Continuous variables were compared between the cities with the *t*-test or Mann–Whitney test. The chi-squared test with continuity correction was used to compare categorical variables. Linear regression models were used to compare spirometry variables between Zabrze and Gdynia, adjusting for age, sex, weight, height, distance to a major road, the presence of coal- or wood-burning stove, the presence of a heating furnace in an in-house living area, and parents’ smoking status. A *p* < 0.05 was considered statistically significant. R software (Version. 3.6.2, R Foundation for Statistical Computing, Vienna, Austria) was used for all analyses.

## 3. Results

### 3.1. Whole-Life Exposure to Air Pollution

The whole-life exposure to PM_10_, NO_2_, and SO_2_ was higher in Zabrze than in Gdynia, but the whole-life exposure to O_3_ was higher in Gdynia (Table 1).

### 3.2. Cohort Characteristics

There were 258 children from Gdynia and 512 children from Zabrze who had spirometry examinations of grade A or B. The cohorts from both cities were similar in age, sex ratio, weight, and height (Table 2). A greater percentage of children in Zabrze than in Gdynia lived near a major road or had a coal- or wood-burning stove or a heating furnace in a living area. (Table 2). Detailed cohort characteristics are shown in Table 2.

### 3.3. Spirometry Variables and Respiratory or Allergic Symptoms

The mean values for FVC, FEV1, and PEF were significantly higher among children in Gdynia than among children in Zabrze (*p* ≤ 0.032), but the mean FEV1/FVC value was significantly greater among children in Zabrze (*p* < 0.001, Table 3). Results of regression models for all of these spirometry variables were presented in the Supplemental Materials (Supplementary Tables S1–S4). 

The frequencies of seasonal rhinorrhea (*p* = 0.015) or coughing episodes (*p* = 0.022) were significantly higher in Zabrze than in Gdynia (Table 4). The frequency of allergic skin reactions tended to be higher in Gdynia (*p* = 0.067, Table 4). Other respiratory or allergic symptoms had similar frequencies in both cities (Table 4).

## 4. Discussion

This study showed that preadolescent children living in Zabrze, in which the air quality is among the worst in Europe, had significantly impaired lung function, as indicated by their lower FVC, FEV1, and PEF compared to their peers from Gdynia on the Baltic coast. Moreover, children in Zabrze suffered from seasonal rhinorrhea or coughing episodes more often than children in Gdynia. There were no significant differences with respect to the frequencies of other allergic or respiratory symptoms.

The whole-life exposure to PM_10_, SO_2_, and NO_2_ was higher in Zabrze than in Gdynia, which was likely industry-related. These findings are in agreement with historical data on air pollution in Silesia and on the Polish coast. Previous evidence has shown that the air pollutants measured in our study have detrimental effects on respiratory health by inducing oxidative damage, inflammation, compensatory proliferation, and airway remodeling and fibrosis [24,25,26]. Consequently, PM_10_, SO_2_, and NO_2_ all slow down lung development in children, as shown by the reduced FVC or FEV1 with increasing concentrations of these pollutants in ambient air [27,28,29].

Our study indicates that the mixed, chronic air pollution in Silesia may be associated with impaired development in preadolescent children. We observed that the FVC in children from Zabrze was more than 200 mL lower than in their peers from the coastal area (~10%). Similarly, FEV1 was lower by about 100 mL (~5%). These findings are in line with those of previously published works. Gehring et al., in a study among 6–8-year-old children, found that FVC and FEV1 both decreased with increasing concentrations of PM_2.5_, NO_2_, and NO_X_ in ambient air [30]. Likewise, Asgari et al. reported decreased FVC and FEV1 among children living in urbanized areas of Iran, with negative correlations between these two spirometry measures and the concentrations of PM, SO_2_, and NO_2_ in ambient air [10]. Another study carried out among children aged 9–13 years who lived in an industrialized area showed decreased FVC and FEV1 owing to increased NO_X_ concentrations [9]. Similarly, FEV1 and FVC were decreased in children living in urbanized areas of China compared to their peers from rural areas, which was also attributed to the difference in air pollution [31]. Rusconi et al. reported that children exposed to oil refinery pollution, characterized by increased concentrations of SO_2_ and NO_2_, had reduced FEV1 and other spirometry measures compared to children living in an area with less air pollution [32]. We observed that the FEV1/FVC ratios were significantly higher in children from Zabrze than in those from Gdynia. This difference was likely because FVC was reduced by a greater extent than FEV1 in children from Zabrze. This explanation is supported by the finding that PEF, which is a measure of airway obstruction, was significantly lower in children from Zabrze than in children from Gdynia. Previous studies have shown that PEF may decrease due to air pollution in asthmatic children [33], but a similar effect among healthy children was reported only in some studies [34] and not in others [35].

We also found that children from Zabrze reported more frequent episodes of coughing or seasonal rhinorrhea compared to children from Gdynia. Air pollution has been linked to an increased risk of respiratory infections [36], which could explain the increased frequency of coughing episodes in Zabrze. Likewise, the increased frequency of seasonal rhinorrhea could be related to the poor air quality in Zabrze, because air pollution plays a role in the development of allergic rhinitis [37]. Although the frequencies of bronchitis, wheezing, and asthma were similar in both cities, one might expect the lifetime risk of asthma to be higher in Zabrze than in Gdynia [38]. Similarly, reduced FVC in children in Zabrze could increase the risk of restrictive lung diseases later in life. Living in Zabrze was not significantly associated with the risk of allergic skin symptoms. This finding agrees with previous evidence that the association between air pollution and skin allergies is less well-established than the association between air pollution and respiratory symptoms [39].

The limitations of our work need to be mentioned. First, the study was cross-sectional, with measurements taken during one season. A longitudinal study could assess whether the differences in lung function are independent of the season. In addition, a longitudinal study would be able to directly investigate lung development in cohorts of children from both cities. Moreover, the concentrations of air pollutants on the days when the spirometry tests were completed were higher in Zabrze than in Gdynia (Supplementary Table S5). Therefore, a further study should assess whether lung function in children from Zabrze remains lower than in their peers from Gdynia when the air pollution on spirometry days is equal. Ideally, spirometry should be repeated in children from Zabrze and Gdynia at the same time and place (e.g., summer camp), but such a study would be difficult to organize. Second, air pollution was not estimated for individual participants. However, we aimed to investigate the effects of long-term air pollution on lung function by comparing children residing in regions that differed substantially in long-term air quality. The air quality data obtained from the Chief Inspectorate of Environmental Protection (Figure 1) showed that the air quality in the entire Zabrze area was poorer than that in Gdynia, which makes the comparison between the two cities valid. Third, children in Zabrze were more exposed to air pollutants inside their homes (wood-burning stoves, heating furnaces in living areas). However, the differences in spirometry variables between Zabrze and Gdynia were significant after adjusting for these confounders. The use of objective measures of lung function is one of the strengths of our study.

In conclusion, this study showed that lung function measured by spirometry was impaired in children from Zabrze, which was likely due to the poor air quality. Moreover, the frequencies of seasonal rhinorrhea and coughing episodes were significantly higher in Zabrze than in Gdynia. We hope that our findings will motivate efforts to improve air quality in Silesia, because better air quality means better lung function [40].

## Figures and Tables

**Table 1:** Air pollution in Gdynia and Zabrze—whole-life exposure by age group.

Age Group	Pollutant							
	PM_10_, µg/m^3^		SO_2_, µg/m^3^		NO_2_, µg/m^3^		O_3_, µg/m^3^	
	Gdynia	Zabrze	Gdynia	Zabrze	Gdynia	Zabrze	Gdynia	Zabrze
9-year-olds	21.96	50.06	3.81	18.18	16.34	25.16	52.40	42.11
10-year-olds	21.73	50.05	3.71	17.68	16.37	24.86	52.44	41.24
11-year-olds	18.83	49.30	3.55	16.85	16.51	24.42	51.79	40.97
12-year-olds	18.41	49.74	3.49	16.97	16.79	24.60	51.28	41.80
13-year-olds	18.06	50.31	3.42	17.08	16.87	24.65	50.78	42.80
14-year-olds	17.59	50.68	3.41	16.91	16.99	24.64	50.98	42.81
15-year-olds	17.16	50.34	3.30	16.07	16.44	24.28	50.99	43.37

Values show means for the whole lifespan depending on age.

**Table 2:** Cohort characteristics.

Variable	Gdynia (*n* = 258)	Zabrze (*n* = 512)	*p*-Value
Age (years), mean ± SD	11.11 ± 1.32	11.23 ± 1.44	0.254
Girls, *n* (%)	123 (47.67)	264 (51.56)	0.308
Height (cm), mean ± SD	152.23 ± 10.41	151.54 ± 11.08	0.406
Weight (kg), mean ± SD	45.45 ± 11.60	45.87 ± 13.43	0.673
BMI (kg/m^2^), mean ± SD	19.38 ± 3.32	19.70 ± 4.17	0.289
Distance to a major road, *n* (%)			<0.001
<100 m	56 (24.24)	173 (37.94)	
100–200 m	64 (27.70)	123 (26.97)	
200–500 m	59 (25.54)	101 (22.15)	
>500 m	52 (22.51)	59 (12.94)	
Coal- or wood-burning stove, *n* (%)	0	11 (2.21)	0.040
Heating furnace in a living area, *n* (%)	17 (6.80)	63 (12.70)	0.014
Smoking parent, *n* (%)	59 (23.51)	144 (28.97)	0.112
Physical activity, *n* (%)			0.011
Not active	2 (0.78)	5 (1.01)	
Irregular	150 (59.76)	238 (48.18)	
Regular	99 (39.44)	251 (50.81)	

**Table 3:** Spirometry variables among schoolchildren in Gdynia and Zabrze.

Variable	Gdynia	Zabrze	*p*-Value *
FVC, mean ± SD (L)	2.42 ± 0.77	2.21 ± 0.73	<0.001
FEV1, mean ± SD (L)	2.17 ± 0.60	2.07 ± 0.67	0.032
FEV1/FVC, mean ± SD	0.91 ± 0.08	0.94 ± 0.07	<0.001
PEF, mean ± SD, (L/min)	259.34 ± 83.90	244.64 ± 81.64	0.018

FEV1, forced expiratory volume during the first second of expiration; FVC, forced vital capacity; PEF, peak expiratory flow. * *p*-values for comparisons between Gdynia and Zabrze are from linear regression models adjusted for age, sex, weight, height, distance to a major road, indoor particulate matter emission sources, and smoking status of parents. Full models are shown in Supplemental Materials.

**Table 4:** Allergic and respiratory symptoms in schoolchildren in Gdynia and Zabrze.

Variable	Gdynia	Zabrze	*p*-Value
All-year rhinorrhea, *n* (%)	15 (6.10)	27 (5.92)	0.925
Seasonal rhinorrhea, *n* (%)	99 (39.60)	241 (48.98)	0.015
Acute allergic reactions, *n* (%)	66 (26.29)	1107 (21.49)	0.140
Dyspnea episodes, *n* (%)	26 (10.53)	58 (11.88)	0.584
Coughing episodes, *n* (%)	56 (22.40)	148 (30.39)	0.022
Wheezing episodes, *n* (%)	23 (9.27)	52 (10.68)	0.552
Skin changes, *n* (%)	108 (43.20)	194 (39.59)	0.345
Allergies (any), *n* (%)	81 (32.66)	134 (27.52)	0.147
Allergic skin reactions, *n* (%)	110 (44.18)	185 (37.23)	0.067
Recurring bronchitis, *n* (%)	46 (18.33)	99 (19.88)	0.612
Wheezing (ever), *n* (%)	32 (12.80)	69 (14.02)	0.646
Asthma diagnosis, *n* (%)	16 (6.56)	37 (7.65)	0.594

## Data Availability

The data presented in this study are available upon request from the corresponding author.

## Supplementary Materials

The following are available online at https://www.mdpi.com/article/10.3390/jcm10112375/s1: Table S1: Regression model for forced vital capacity (FVC, liters). Table S2: Regression model for forced expiratory volume in 1 s (FEV1, liters). Table S3: Regression model for FEV1/FVC. Table S4: Regression model for peak expiratory flow (PEF, liters/min). Table S5: Air pollution on spirometry days.
