# Supplementary material for: Impact of Air Pollution on Lung Function among Preadolescent Children in Two Cities in Poland
Source: J Clin Med. 2021 May 28;10(11):2375. doi: 10.3390/jcm10112375 (PMC8198305; doi:10.3390/jcm10112375)
Supplement: Supplementary file 1 [file jcm-10-02375-s001.zip › jcm-1201453-supplementary.pdf]

Supplementary Files

Table S1. Regression model for forced vital capacity (FVC, liters).

	Estimate	Std. Error	t value	Pr(>|t|)
(Intercept)	−2.872	0.372	−7.705	<0.001
Age (years)	0.912	0.021	4.184	<0.001
Sex (male_	0.083	0.043	1.913	0.056
Weight (kg)	0.011	0.002	4.732	<0.001
Height (cm)	0.024	0.003	6.844	<0.001
City (Zabrze vs. Gdynia)	−0.186	0.046	−4.010	<0.001
Distance to major road (category)	0.011	0.020	0.581	0.561
Heating furnace in living area (yes vs. no)	0.013	0.073	0.179	0.858
Smoking parent (yes vs. no)	0.009	0.050	0.184	0.854
Coal- or a wood-burning stove	−0.274	0.290	−0.945	0.344

Residual standard error: 0.5628 on 672 degrees of freedom (88 observations deleted due to missingness); Multiple R-squared: 0.4361, Adjusted R-squared: 0.4285; F-statistic: 57.73 on 9 and 672 DF, p-value: < 0.00000000000000022.

Table S2. Regression model for forced expiratory volume during the first second of expiration (FEV1, liters)

	Estimate	Std. Error	t value	Pr(>|t|)
(Intercept)	−2.826	0.314	−8.999	<0.001
Age (years)	0.086	0.018	4.697	<0.001
Sex (male_	0.051	0.036	1.415	0.157
Weight (kg)	0.007	0.001	3.544	<0.001
Height (cm)	0.024	0.002	8.077	<0.001
City (Zabrze vs. Gdynia)	−0.084	0.039	−2.148	0.032
Distance to major road (category)	0.012	0.017	0.743	0.457
Heating furnace in living area (yes vs. no)	0.005	0.061	0.089	0.929
Smoking parent (yes vs. no)	−0.007	0.042	−0.188	0.851
Coal- or a wood-burning stove	−0.063	0.244	−0.261	0.794

Residual standard error: 0.4742 on 672 degrees of freedom (88 observations deleted due to missingness); Multiple R-squared: 0.4568, Adjusted R-squared: 0.4495, F-statistic: 62.79 on 9 and 672 DF, p-value: < 0.00000000000000022.

Table S3. Regression model for FEV1/FVC.

	Estimate	Std. Error	t value	Pr(>|t|)
(Intercept)	0.850	0.047	17.803	<0.001
Age (years)	0.000	0.002	0.016	0.987
Sex (male_	−0.005	0.005	−1.076	0.282
Weight (kg)	−0.001	0.000	−4.221	<0.011
Height (cm)	0.008	0.000	1.852	0.064
City (Zabrze vs. Gdynia)	0.029	0.005	4.913	<0.001
Distance to major road (category)	−0.000	0.002	−0.205	0.837
Heating furnace in living area (yes vs. no)	−0.001	0.009	−0.197	0.843
Smoking parent (yes vs. no)	−0.005	0.006	−0.859	0.390
Coal- or a wood-burning stove	0.077	0.037	2.074	0.038

Residual standard error: 0.07212 on 672 degrees of freedom (88 observations deleted due to missingness); Multiple R-squared: 0.06711, Adjusted R-squared: 0.05461; F-statistic: 5.371 on 9 and 672 DF, p-value: 0.0000004056.

Table S4. Regression model for peak expiratory flow (PEF, liters/min).

	Estimate	Std. Error	t value	Pr(>|t|)
(Intercept)	−259.955	45.213	−5.749	<0.001
Age (years)	9.858	2.646	3.726	<0.001
Sex (male)	3.422	5.277	0.649	0.516
Weight (kg)	0.666	0.294	2.267	0.023
Height (cm)	2.448	0.428	5.714	<0.001

City (Zabrze vs. Gdynia)	−13.270	5.632	−2.356	0.018
Distance to major road (category)	3.623	2.500	1.449	0.147
Heating furnace in living area (yes vs. no)	−0.000	0.009	−0.197	0.843
Smoking parent (yes vs. no)	−0.005	0.006	−0.859	0.390
Coal- or a wood-burning stove	−3.851	35.221	−0.109	0.912

Residual standard error: 68.27 on 672 degrees of freedom (88 observations deleted due to missingness); Multiple R-squared: 0.3039, Adjusted R-squared: 0.2945, F-statistic: 32.59 on 9 and 672 DF, p-value: < 0.00000000000000022.

Table S5. Air pollution on spirometry days.

**Pollutant**	**Gdynia**	**Zabrze**	**p-value***
PM_{10}, μg/m^3	12.99 ± 1.46	23.90 ± 4.24	<0.001
SO_2, μg/m^3	1.22 ± 0.18	6.12 ± 1.51	<0.001
NO_2, μg/m^3	11.87 ± 4.78	16.31 ± 1.98	<0.001
O_3, μg/m^3	54.79 ± 14.35	74.41 ± 7.94	<0.001

Values show means±standard deviations; *,t-test.
